# Revisiting Genetic Influence on Mercury Exposure and Intoxication in Humans: A Scoping Review

**DOI:** 10.3390/toxics11120967

**Published:** 2023-11-29

**Authors:** Maria Elena Crespo-Lopez, Jean Ludger Barthelemy, Amanda Lopes-Araújo, Leticia Santos-Sacramento, Caio Gustavo Leal-Nazaré, Isabela Soares-Silva, Barbarella M. Macchi, José Luiz M. do Nascimento, Gabriela de Paula Arrifano, Marcus Augusto-Oliveira

**Affiliations:** 1 Laboratório de Farmacologia Molecular, Instituto de Ciências Biológicas, Universidade Federal do Pará, Belém 66075-110, PA, Brazilletisacramentolfm@gmail.com (L.S.-S.); caiokgustavo@gmail.com (C.G.L.-N.);; 2 Laboratório de Neuroquímica Molecular e Celular, Instituto de Ciências Biológicas, Universidade Federal do Pará, Belém 66075-110, PA, Braziljlmn@ufpa.br (J.L.M.d.N.)

**Keywords:** methylmercury, susceptibility, SNP, polymorphism, Amazon, health

## Abstract

Human intoxication to mercury is a worldwide health problem. In addition to the type and length of exposure, the genetic background plays an important role in mercury poisoning. However, reviews on the genetic influence in mercury toxicity are scarce and not systematic. Therefore, this review aimed to systematically overview the most recent evidence on the genetic influence (using single nucleotide polymorphisms, SNPs) on human mercury poisoning. Three different databases (PubMed/Medline, Web of Science and Scopus) were searched, and 380 studies were found that were published from 2015 to 2022. After applying inclusion/exclusion criteria, 29 studies were selected and data on characteristics (year, country, profile of participants) and results (mercury biomarkers and quantitation, SNPs, main findings) were extracted and analyzed. The largest number of studies was performed in Brazil, mainly involving traditional populations of the Tapajós River basin. Most studies evaluated the influence of the SNPs related to genes of the glutathione system (GST, GPx, etc.), the ATP-binding cassette transporters and the metallothionein proteins. The recent findings regarding other SNPs, such as those of apolipoprotein E and brain-derived neurotrophic factor genes, are also highlighted. The importance of the exposure level is discussed considering the possible biphasic behavior of the genetic modulation phenomena that could explain some SNP associations. Overall, recommendations are provided for future studies based on the analysis obtained in this scoping review.

## 1. Introduction

Mercury is a toxic heavy metal that has serious effects on human health and the environment [1,2,3]. It is currently classified among the top three priority substances of concern for public health due to its ubiquitous presence and deleterious consequences [4]. Mercury is released into the environment from diverse anthropogenic activities; artisanal and small-scale gold mining (ASGM) is the mainly responsible for emissions into the air [5,6]. Additionally, mercury mobilization and human exposure is facilitated by anthropogenic alterations of the environment, such as fires and dams [3,7,8,9]. As a result, a wealth of evidence from various regions around the world showcases the detrimental effects of this metal on the delicate balance between human activities and the environment [10,11,12,13]. Chronic exposure to mercury in humans frequently occurs through the intake of contaminated food, especially fish, which is an important source of protein for many vulnerable populations worldwide [14,15]. 

The central nervous system (CNS), mainly the brain, is the main target where mercury accumulates, causing neurological symptoms including tremors, behavioral changes, memory loss, irritability and insomnia, neuropathy, and hearing loss [6,16]. Moreover, recent evidence demonstrates that chronic exposure to mercury can also damage other systems, such as the cardiovascular system, leading to deleterious consequences including hypertension and metabolic syndrome [17,18,19].

Unfortunately, once the symptoms of mercury intoxication are evident, recovery is unlikely, even when mercury chelators are used to decrease the metal levels in the body [20,21]. Therefore, the best strategy for public policies seems to be the prevention of mercury exposure and the identification of individuals at high risk. However, despite the worldwide exposure to mercury and the increased emissions in the last years, our knowledge on how chronic exposure and intoxication is modulated by the individual susceptibility is scarce. In the last decades, the advances in genetics and sequencing have revolutionized the diagnosis and treatment of many diseases. It is now possible to determinate the individual risk of developing some diseases, including rare diseases and cancers, based on the epidemiological association between genetic markers and the development of these diseases. Therefore, personalized medicine has made great strides, leading to more effective treatment [22,23].

Genetic background is an important factor in heavy metal poisoning [24,25]. Single base polymorphisms (SNPs) of genes encoding differences in proteins play a key role in the modulation of both the toxicokinetics (absorption, distribution, metabolism and excretion) and toxicodynamics (interactions with molecular targets and adverse effects) of mercury [26,27,28,29]. Studies using SNPs allow the identification of alleles that increase human susceptibility to mercury intoxication and/or exposure, consequently identifying the most vulnerable individuals/populations in terms of genetic susceptibility. Unfortunately, review articles on the influence of genetic variation on mercury poisoning are relatively scarce [24,30] and, to date, no systematic search in different databases has been carried out. Therefore, this scoping review followed international recommendations of quality [31] to present an overview of the most recent evidence on the influence of genetic variation (SNPs) in the human susceptibility to mercury, identifying the limitations of studies and providing insights for future research.

## 2. Materials and Methods

The present scoping review followed the PRISMA guidelines [31]. The search was performed using three databases (PubMed/Medline, Web of Science and Scopus), with the terms Polymorphism* and Mercury* (in the title, abstract and keywords fields). The search included studies from 2015 to 2022 (the period not covered by previous reviews). Different inclusion/exclusion criteria were applied to the screening: original, experimental and peer-reviewed study (1), epidemiological evaluation of populations exposed to mercury (2), the presence of mercury quantitation confirming the exposure (3) and SNP analysis (4) in humans. No geographical or language restrictions were applied. These criteria were checked first in the title and abstract and then in the entire article when necessary. When a study was not available, a copy was requested from the authors. The search and studies were independently reviewed by three co-authors and a final assessment was made by a fourth co-author to ensure that the criteria were met. The following data were extracted from the selected studies: (i) country where the study was performed, (ii) year of publication, (iii) profiles of the participants, (iv) quantitation of mercury, (v) type of mercury biomarker, (vi) polymorphisms, (vii) purpose of the study and (viii) the main findings. 

## 3. Results and Discussion

### 3.1. Results of the Screening Process

**Figure 1:**
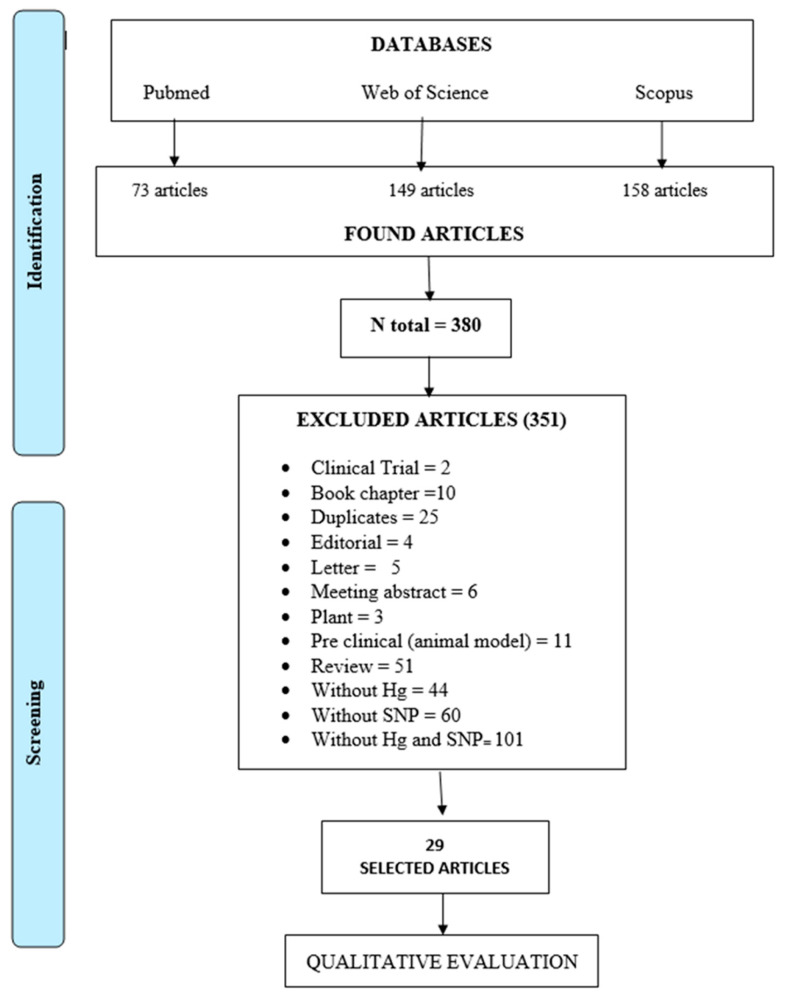
Flowchart of the search and screening process performed in this scoping review.

A total of 380 studies were found in the three databases (Figure 1). Of them, 351 studies were excluded, including 2 clinical trials, 10 book chapters, 25 duplicate studies, 4 editorials, 5 letters, 6 meeting abstracts, 3 studies in plants, 11 pre-clinical (animal model) studies, 51 review papers, 44 studies without mercury quantitation, 60 studies without SNP analysis, and 101 studies without both mercury quantitation and SNP analysis. Therefore, a total of 29 studies were finally selected (Figure 1).

### 3.2. Origin of the Studies and Populations

**Figure 2:**
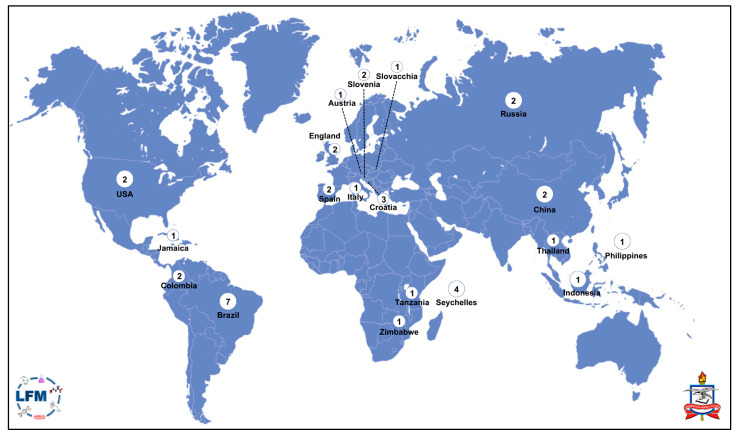
World map with the number of studies related to mercury and genetic polymorphisms performed in each country during 2015–2022.

The selected studies, according to the inclusion/exclusion criteria, were performed in 19 countries (Figure 2), most of them in Brazil (seven), which is expected since it is one of the five countries that most emit mercury into the air and the third in emissions from ASGM according to the United Nations Environment Programme [5]. Interestingly, most studies involved the traditional populations of the Tapajós River basin, the region with the highest density of ASGM in the Amazon [3,16]. Currently, the Amazon is one of the largest global contributors to the emissions of mercury into the atmosphere. About 80% of all emissions from South America originate from this region [32]. It is estimated that more than 200 tons of mercury are released annually from the Amazon, although this quantity could be greatly underestimated [3,7]. As with many populations in the Amazon, the majority of residents in the Tapajós River basin are vulnerable populations who are located in remote regions with limited access to healthcare and higher education [33]. Amazonian populations usually show a trihybrid ancestry with three major contributors (Amerindian, European and African), with European origin having the highest contribution [34,35,36]. Interestingly, a significant higher Amerindian contribution has been recently associated with a higher susceptibility to mercury [36]. This is a relevant fact, indicating that more studies on populations from the Southern Hemisphere and their characteristics are needed. Although the countries that most emit mercury from ASGM (Indonesia, Perú and Brazil) are located in the Southern Hemisphere [5], our current knowledge about human exposure and intoxication is mainly from cohorts from developed countries, such as NHANES (USA), ALSPAC (UK) or REGARDS (USA). Especially worrying is the low number of studies from the sub-Saharan region, despite this region accounting for 16% of the global emissions of mercury (with up to 80% due to ASGM). In this study, we found only one study with populations from Africa (Tanzania and Zimbabwe) and only two with populations from Asia (Thailand, Philippines and Indonesia). Only three studies included populations of more than one country: Austria and Slovakia [37], Slovenia and Croatia [38], and the Philippines, Indonesia, Tanzania and Zimbabwe [27]. However, these studies did not analyze the ancestry, which would be highly recommended, especially for the latter study by Kolbinger et al. (2019), which included populations from three different continents [27].

### 3.3. Other Features of the Studies: Year of Publication and Biomarkers

**Figure 3:**
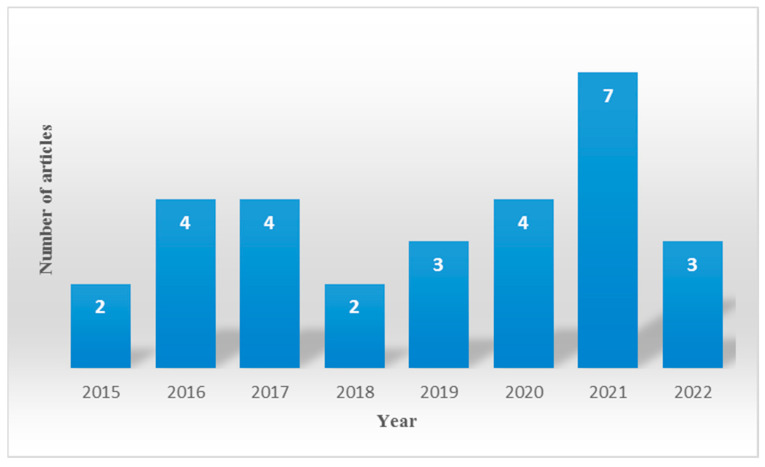
Number of studies related to genetic influence on human exposure to mercury and intoxication published during 2015–2022, grouped per year. Details of the studies can be found in Table 1.

Interest in the genetic influence in mercury intoxication and exposure has increased during the last years, showing a growing tendency in the number of studies (Figure 3). Considering the increase in mercury emissions worldwide, it is very promising that the scientific community increasingly contributes to improving our knowledge on this matter, elucidating new polymorphisms involved in human susceptibility to mercury that support the early identification of individuals at risk and the implementation of protective policies and preventive strategies.

The most frequent matrixes (biomarkers) used to assess mercury exposure in human populations were hair and blood (Table 1), which are in agreement with the main pathways of human exposure via contaminated food intake, especially fish and rice. Only one work included mercury quantitation in toenails to assess the exposure [39], although the validity and epidemiological relevance of this biomarker is still being discussed. This issue is very important since no association was found between the SNPs and the mercury in toenails [39]. Consequently, the appropriate choice of the biomarker is essential for biomonitoring and assessing human exposure to mercury [33]. The use of hair, for example, facilitates field work and sampling, as hair is easy to collect, store at room temperature, and manipulate. Although blood is also a reliable marker of exposure, the collection of blood is invasive and mercury is significantly less concentrated in this matrix compared with hair so requires more sensible techniques.

**Table 1:** Main characteristic of the extracted data obtained in the systematic search. Results of populations showing mean/median levels of exposure above those (9.2 µg/L of blood mercury, 2.3 µg/g of hair mercury, and 50 µg/g creatinine of urine mercury) equivalent to the maximum exposure recommended by the World Health Organization [3,7,40] are highlighted in gray.

Population	Exposure				Gene (SNPs)	Study
	Main Type	N (Women, Men)	Matrix	Total Hg (Mean or *Median* *)		
Adults	Environmental	395 (188, 207)	Blood	*39.8 µg/L*	GSTM1 (deletion), GSTT1 (deletion), GSTP1 (rs1695), GCLM (rs41303970), GCLC (rs17883901), GPX1 (rs1800668), ALAD (rs1800435), VDR (rs1544410), MDR1 (rs2032582)	[41]
		113 (50, 63)		7.0 µg/L (plasma)	eNOS (rs11771443, rs1799983, VNTR 4a/4b)	[42]
		889 (498, 391)		1.40 μg/L	PON1 (rs662)	[43]
		436 (152, 284)		6.31 µg/L	MT1A (rs 8052394)	[44]
		200 (200, 0)		1.8 µg/kg (erythrocytes)	MT1A (rs11640851), MT4 (rs11643815), HFE (rs1800562, rs1799945), VDR (rs1544410), ALAD (rs1800435), GSTP1 (rs1695, rs1138272), GCLC (rs17883901), GCLM (rs41303970), ABCB1 (rs2032582, rs1128503, rs2032582), ABCB11 (rs2287622, rs497692), ABCC1 (rs246221), ABCC2 (rs717620, rs2273697), ABCG2 (rs2231142), UGT2B15 (rs1902023)	[37]
		149 (149, 0)	Hair	0.6 μg/g	GPX1 (rs1800668), GSTM1(deletion)	[45]
		823 (521, 302)		*4.84 µg/g*	APOE (rs429358, rs7412)	[36]
		200 (109, 91)		6.6 µg/g	TNF-α (rs1799964, rs1799724, rs1800629), IL6 (rs1800795), ALAD (rs1800435), GSTP1 (rs1695), VDR (rs2228570), MMP2 (rs2285053)	[46]
		2562 (1130, 1432)	Toenails	*0.066 μg/g*	MTF1 (rs12751325, rs3748682), SLC7A8 (rs11624694, rs17183863), MT4 (rs11643815, rs17285449, rs7186103), MMP2 (rs17859821, rs2576550, rs11859163, rs34373154)	[39]
	Environmental and occupational	380 (142, 238)	Hair Blood Urine	0.62 µg/g 3.75 µg/L 1.32 µg/L	GCLC (rs17883901), GLRX2 (rs912071), TXNRD2 (rs5748469), MT1B (rs7191779, rs8052334), MT1M (rs2270836), MT4 (rs11643815), SLC7A7 (rs2281677), SLC43A2 (rs4790732), DNMT1 (rs2228613), GCLC (rs17883901), GSTA4 (rs367836), GSTP1 (rs1695, rs1138272), TXNRD2 (rs5748469), MT1M (rs2270836), MT4 (rs11643815), ABCB1 (rs9282564), SLC22A8 (rs4149182), SLC43A2 (rs4790732), DNMT1 (rs2228613), GPX6 (rs6413428), SEPN1 (rs7349185), SEPN1 (rs2294228), SEPHS2 (rs1133238), SEPP1 (rs3877899), TXNRD3 (rs3108755), ATP7B (rs1801243), SLC22A6 (rs4149170), MTHFR (rs2274976), HBS1L (rs4895441)	[47]
	Occupational	180 (0, 180)	Blood	18.67 µg/L	GSTM1 (deletion) GSTT1 (deletion)	[48]
		120 (0, 120)	Blood Urine	In 1987 (blood): 74.93 μg/L In 2005 (urine): 6.22 µg/L, 0.21 µg/L 18.22 µg/L	HSPA1B (rs1061581), HSP1A1 (rs1043618), HSP1AL (rs2227956)	[49]
		968 (391, 577)	Urine	10 μg/g creatinine	ABCC2 (rs1885301, rs717620, rs2273697)	[27]
		281 (121, 160)	Blood Urine Hair	*7 µg/L* *3.8 µg/g creatinine* *0.8 µg/g*	ABCB1 (rs1202169), ABCC2 (rs1885301), SLC22A6 (rs 4149170), SLC22A8 (rs 4149182)	[50]
		281 (121, 160)	Blood Urine Hair	7 µg/L 3.8 µg/g creatinine 0.8 µg/g	GCLC (rs 1555903), GCLM (rs41303970), GSS (rs3761144), GSTA1 (rs3957356), GSTP1 (rs 4147581)	[29]
Mothers and children	Environmental	1331	Hair	3.9 µg/g	ABCC1 (rs215088, rs1292798, rs1107529, rs246241, rs212093, rs11075290); ABCC2 (rs2273697, rs717620, rs2756103, rs7393105, rs2273697); ABCB1 (rs2032582, rs2235035, rs1027458, rs1202169, rs1202171, rs1027649, rs2032582).	[51]
		1688	Hair U cord	0.361 µg/g 0.002 µg/g	APOE (rs429358, rs7412)	[38]
		2898	Blood Hair U cord	18.22 μg/L 3.87 µg/g 34.48 μg/L	GCLM (rs 41303970), GCLC (rs 761142), GSTP1(rs 1695)	[52]
		562	Blood Hair Serum Placenta U cord Cord serum	3.54 μg/L 0.88 µg/g 0.78 μg/L 9.49 μg/kg 5.85 μg/L 0.61 μg/L	MT2A (rs 28366003)	[53]
		436	Blood Hair Milk U cord Urine	0.002 μg/g 0.51 µg/g 0.14 ng/g 0.003 μg/g 0.74 μg/L	APOE (rs 429358, rs7412)	[54]
		344	Hair	*0.99 μg/g (mother)* *1.02 µg/g*	GCLC (rs 17883901), GCLM (rs41303970), GPX1 (rs1050450), GSTA1 (rs3957356), GSTP1 (rs1695), MT1M (rs2270836, rs9936741), MT2A (rs10636), MT4 (rs 11643815)	[55]
		946	U cord	35 µg/L	ABCB1 (rs2032582, rs10276499, rs1202169), ABCC1(rs11075290 e rs215088), ABCC2 (rs717620)	[56]
		2639	M hair U cord	NC1- Seychelles: 5.8 µg/g 39.3 μg/L NC2-Seychelles: 3.9 µg/g INMA-Spain: 11.3 μg/L PHIME-Italy: 1.0 µg/g 5.6 μg/L	CYP3A7 (rs2257401), CYP3A5 (rs776746), CYP3A4 (rs2740574)	[57]
Children	Environmental	2172	U cord	2.70 μg/L	ABCA1 (rs4149268, rs3890182), TF (rs3811647), PON1 (rs662), BDNF (rs2049046), PGR (rs1042838), SOD2 (rs5746136), MT1M (rs2270836)	[58]
		532	Blood	1 µg/L	GSTP1 (rs 1695), GSTT1 (deletion), GSTM1 (deletion)	[59]
		103	Hair	7.0 µg/g	ALAD (rs 1800435)	[60]
		403		0.89 µg/g	PON1 (rs662; rs705381), BDNF (rs1519480, rs7934165, rs6265, rs12273363, rs7103411), APOA4 (rs5110) APOE (rs7412), GSTP1 (rs1695)	[28]
		412	Hair at 9 years old U cord	*Female:* *1.0 μg/g* *11.0 μg/L* *Male:* *0.8 μg/g* *10.7 μg/L*	BDNF (rs12273363, rs7934165, rs7103411, rs1100104, rs6265, rs925946)	[61]
		466	Urine	*1.06 μg/g creatinine* *(n = 238)*	BDNF (rs6265, rs2883187, rs7124442)	[62]

U cord: Umbilical cord; M hair: Maternal hair. * In the table, median levels are showed in italics and mean levels as normal letters.

Most studies (10) included participants of both genders, women and men; only four included just women (Table 1). Interestingly, seven studies included paired mothers and children, with polymorphisms influencing child development (including neuropsychological development, cognitive deficits and motor impairments) (Table 1).

### 3.4. Analysis of Genetic Susceptibility to Mercury Exposure and Intoxication

Overall, the most studied SNPs in human populations exposed to mercury were those related to the metal detoxification, such as those of the glutathione system (glutathione S-transferase (GST) genes (11 studies), glutathione peroxidase (GPX) genes (4 studies) and the glutamate–cysteine ligase catalytic (GCLC) and regulatory (GCLM) subunit genes (6 studies)), the ATP-binding cassette (ABC) transporters (7 studies) and the metallothionein (MT) proteins (6 studies) (Table 1). The only study analyzing the CYP3A proteins, the main superfamily proteins responsible for the metabolism of drugs and other xenobiotics, found no association with umbilical cord mercury [57]. Other interesting works were devoted to genes involved in the cellular mechanisms of mercury toxicity, such as neurodegeneration (apolipoprotein E (APOE) (six studies) and brain-derived neurotropic factor (BDNF) (4 studies) genes) and oxidative stress (paraoxonase 1 (PON1) and nitric oxide synthase (NOS) genes, among others). Table 2 shows some highlights of the main statistically significant associations found in these studies.

The glutathione system is the main mercury detoxification pathway [63]. The GST Pi 1 (GSTP1) protein is one of the most expressed members of the GST family and catalyzes the conjugation of glutathione to mercury [30]; it is found in many cells and organs, such as in the erythrocytes, placenta, lung, brain, muscle and liver, among others. The rs1695 polymorphism of the GSTP1 gene shows adenine instead of guanine at position 313, resulting in the amino acid exchange of valine for isoleucine at position 105 in the protein and meaning a decrease in the enzymatic activity [64,65]. However, conflicting results are found. In epidemiological studies, the minor allele G was associated with higher hair mercury [55], lower blood mercury [47] or no change in mercury levels but decreased cognitive and psychomotor development in children of mothers carrying this allele [52]. Interestingly, with an increase in mercury levels, children carrying the rs1695 GA or GG alleles scored worse on problems such as anxiety, depression and somatic complaints than children with the AA genotype [28]. Consequently, in addition to the possible effects on mercury detoxification, the rs1695 SNP may show influence on toxicodynamic mechanisms, with synergistic effects to those of the metal. It is essential that future in vitro and in vivo studies focus on better understanding these mechanisms and consider the exact context of the exposure (doses of translational relevance, type (acute or chronic) and pathway (oral or respiratory)), as suggested elsewhere [66].

To detoxify mercury, the GST enzymes need reduced glutathione (GSH), which depends on the activities of two other enzymes, GPX and glutathione reductase. GPX is a selenoprotein that reduces peroxides (hydrogen peroxide and lipid peroxides) by oxidizing GSH to GSSG. GPX1 is the more abundant isoenzyme of this family and is found in the cytoplasm of almost all mammalian tissues. Two SNPs of the GPX1 gene, rs1050450 and rs1800668, have been studied regarding possible mercury level modulations (Table 1); however, only the presence of the rs1050450 minor allele T was significantly associated with lower hair mercury in low fish consumers [55]. Interestingly, this effect was not observed either in high fish consumers or children [55], pointing to a relatively low influence of the SNP and/or a possible biphasic phenomenon depending on the mercury doses.

Other important polymorphisms related to the glutathione system are the deletions in GSTM1 and GSTT1 genes due to their influence in the synthesis of glutathione [67]. The deletion of GSTM1 has already been associated with increased mercury concentrations in human blood [41]. Theses deletions cause the null activity of the glutathione-S transferase enzyme, decreasing the conjugation of glutathione to mercury and consequently decreasing mercury elimination [41].

Looking at a previous step in the glutathione system, polymorphisms related to glutathione synthesis have been associated with a modulation in exposure levels. For example, the SNP GCLC rs761142 TT was associated with an increase in maternal hair mercury levels, and this association is amplified when combined with GCLM rs41303970 CC; thus, double homozygotes (TT+CC) had even higher hair mercury levels [52]. The cellular mechanisms of this combined effect on increasing mercury levels are still not fully understood. Interestingly, the SNP GCLC rs761142 TT was also associated with impaired motor development in children [52]. Conversely, the opposite effect for mercury accumulation was described for carriers of the allele T of the SNP GCLC rs17883901, which showed lower hair mercury levels [55].

Another important system that may play a role in mercury detoxification involves the ABC transporters, a family of membrane proteins [68]. Interestingly, the SNPs ABCC1 (rs11075290, rs212093 and rs215088), ABCC2 (rs717620) and ABCB1 (rs10276499, rs1202169 and rs2032582) were associated with the modulation of maternal mercury hair levels [37,51]. However, the exact mechanisms and influence of these SNPs on mercury distribution, particularly during pregnancy, are not totally understood [37,51]. For example, the ABCC1 rs246221 C allele was associated with a higher rate of placental mercury transfer [37] and the SNP rs11075290 of the same gene was correlated with decreased umbilical cord mercury (carriers of the T allele showed lower cord mercury levels) [56]. The latter allele was associated with an increased expression of the ABCC1 gene, which could mean a higher mercury detoxification rate [56]. However, the associations between ABC SNPs, mercury distribution and neurodevelopment are still controversial. For example, children born to mothers with the rs11075290 CC genotype (with lower hair mercury) showed impaired neurodevelopment, as evaluated by the Mental Development Index (MDI) and Psychomotor Development Index (PDI) [51]. The worse performance on neurological tests associated with the SNPs ABCC2 (rs2273697, rs1885301) could be hypothetically explained by mercury accumulation due to the decreased protein expression; alternatively, these SNPs could non-functional and the observed effects may be linked to other polymorphisms that are not yet known and may be truly functional [27]. Additional studies are necessary to elucidate the exact role of the ABC SNPs in mercury detoxification and how this role is related to the neurodevelopmental outcomes.

The metallothionein gene (MT) SNPs are associated with the modulation of both the body burden and neurological outcomes caused by the metal. For example, the MT1M rs9936741 T allele was associated with higher Hg levels in mothers [55] and the MT1A rs8052394 G allele was associated with decreased attention, memory and visuospatial/executive ability in elderly individuals exposed to higher mercury levels [44]. MTs are low-weight metal-binding and cysteine-rich proteins that play an important role in both metal detoxification, such as mercury elimination, and antioxidant defense [69]. The cysteine residues of these proteins bind mercury, decreasing the free form of the metal and, consequently, its availability. Additionally, these proteins show antioxidant effects, protecting the cells and tissues against mercury toxicity [69]. However, the exact mechanisms, which may explain the significant modulation of mercury levels by SNPs such as MT1M rs9936741 and MT1A rs8052394, are still unknown [55]. Of note, as observed for other genes, the modulation found for MT1M was only observed with low exposure and was not detected in the high-exposure group [55].

### 3.5. Insights and Recommendations

Overall, the conflicting results found for the relationship between mercury and some of these SNPs could be explained, at least partially, by the context of the exposure level. Since a possible biphasic behavior has already been described for mercury-related phenomena [3,36,70], it is important to pay attention to whether the mean/median level of the exposed population is above or below the maximum recommended limits. Furthermore, the strict classification as only occupational exposure has to be cautiously interpreted, since the individuals are probably being exposed to mercury by different pathways simultaneously due to the ubiquity of this metal. A good example of this fact is the work by Parajuli et al. [47], who detected simultaneous exposures (both occupational and environmental) in dental professionals recruited at an annual meeting, demonstrating the importance of additionally evaluating hair mercury (main biomarker of environmental exposure) in the occupationally exposed population. Compared with hair or urinary mercury, blood mercury provides less information as a biomarker of the exposure source (and is probably more variable due to the lower concentration and the probable influence of the time of the day in the sample collection, among other confounders). Interestingly, in the latter work [47], despite all participants being dental professionals, the quantitation of mercury was suggestive of a higher contribution from environmental exposure and the genetic modulation was mainly associated with hair mercury and fish intake (Table 1 and Table 2). In Colombian miners, hair mercury has already been used as a confounder for the effects of methylmercury due to fish consumption [29,50]. In this cohort, ABCB1, SCL22A8 and GCLM were significantly associated with a higher clearance rate of urinary mercury [29,50]. Unfortunately, the possible interference of environmental exposure was not considered in the other studies of occupationally exposed populations (Table 1). 

The World Health Organization (WHO) recommends a maximum weekly intake of approximately 100 micrograms of methylmercury [40], which is equivalent to 2.3 µg/g and 9.2 µg/L of hair and blood mercury, respectively [3,66]. In our review, all studies exceeding the latter levels of exposure were performed in populations from the Southern Hemisphere; in fact, the median level of exposure found in these studies was approximately 5 µg/g of mercury hair, i.e., about five times the exposure found in the studies carried out in the Northern Hemisphere (Table 1). 

In addition to this significant difference in the exposure context, another essential issue that is hardly taken into account in genetic studies is the different genetic backgrounds of the populations of the two Hemispheres. Indigenous ancestry, for example, has been already associated with a higher prevalence of apolipoprotein E (ApoE, for the protein; APOE, for the gene) 4 allele and the absence of the APOE2 allele. ApoE is the main lipid transporter that is highly expressed in the brain [26]. It plays an essential role in lipid metabolism and neuronal repair [26]. The presence of APOE4 has its main effects in the CNS, creating a deleterious scenario with cellular mechanisms of damage (oxidative stress, mitochondrial dysfunction, blood–brain barrier impairment, neuroinflammation, etc.) [26]. Many of these mechanisms are also induced by mercury intoxication; possible synergic effects between both conditions are hypothesized [26]. Epidemiologically, the presence of APOE4 is associated with signs such as anxiety, depression, somatic complaints and social, thought and attention problems in exposed children [28]. In addition to the worse scenario for the CNS provided by the APOE4 presence, highly exposed individuals carrying the latter genotype accumulated significantly more mercury than those with the ApoE2 protein [36]. Interestingly, this phenomenon was not detected in low-exposed individuals, pointing to the importance of monitoring this allele, especially in the Southern Hemisphere populations (where higher proportions of indigenous ancestry can be additionally found). This different modulation according to the exposure level could explain, at least in part, the conflicting results found in the association between mercury effects on child development and the APOE4 genotype in the other three studies obtained in our search (all of them carried out in European populations presenting median/mean mercury levels below 1 µg/g) [28,38,54]. Interestingly, the different influence of genetic modulation according to the exposure level related to fish intake was also demonstrated for dental professionals in the USA for other SNPs (MT1M rs2270836, MT4 rs11643815, ATP7B rs1061472 and rs732774, BDNF rs6265 and GCLC rs138528239) [47]. 

Unfortunately, the number of studies carried out in the highly exposed populations of the Southern Hemisphere is insufficient to reach a conclusion about the influence of some SNPs that presents limited/conflicting results, such as the aminolaevulinic acid dehydratase (ALAD) gene (the SNP rs1800435 presented no association with mercury levels in three of the four studies analyzing this SNP); therefore, it is urgent to conduct additional studies to increase our knowledge of these populations.

Among all the SNPs used to analyze the possible genetic modulation of mercury toxicity, an interesting case is the SNPs related to the brain-derived neurotrophic factor (BDNF) gene that has recently been used to evaluate impaired development associated with mercury exposure in children (Table 1). Compared with adults, children are especially susceptible to the effects of mercury due to their developing CNS, and the BDNF gene has been proposed as an effect modifier, i.e., influencing the toxicodynamic effects of the metal (not the mercury levels). BDNF plays an essential role in neuronal survival and growth, neurotransmitter modulation and neuronal plasticity, which are essential for learning and memory. In the brain, mercury-induced BDNF expression in astrocytes protects neurons against metal toxicity [1,6,71]. Although in vivo studies on BDNF and mercury in recent years are not scarce, concern has been raised about the translational meaning of the doses used in these studies [7,66,71], which has prevented the description of unspecific mechanisms. Future studies must provide the exposure context of their conclusions according to the translational meaning of that exposure [66].

Among some metals, mercury was the only one significantly associated with the risk of dyslexia in children, and the BDNF SNP rs6265 modulates this association [62]. The TT genotype of this SNP significantly increased the risk of dyslexia in highly exposed children (interestingly, this effect was not detected in children who had low exposure) [62]. Additionally, the latter genotype in exposed children significantly increased the Attention Deficit Hyperactivity Disorder (ADHD) Index, which measures child behavior, particularly related to inattention and hyperactivity [28]. Other BDNF SNPs, such as the CC genotypes of rs1519480, rs12273363 and rs7103411 and the GG genotype of rs7934165, were also associated with a higher ADHD Index [28], showing the importance of this gene as an attention-related effects modifier in exposed children. Some of these SNPs additionally increased the risk of internalizing problems such as anxiety, depression and somatic complaints [28]. In addition, recent evidence points to a possible role for the modulation of the effect of mercury via the BDNF gene on children’s sexual development [61]; however, additional studies are necessary to confirm and understand this possible association.

Other mercury effects that need more attention are those related to the deleterious consequences on the cardiovascular system. Two recent and elegant systematic reviews and meta-analyses [70,72] have demonstrated that hair mercury levels above 2 μg/g (approximately 8 μg/L of blood mercury and a weekly consumption of 1.4 μg MeHg/Kg b.w., according to the equivalencies proposed elsewhere [3]) are associated with: (i) a 59% increase in the odds ratio for hypertension and (ii) a significant increase in the risk of fatal and non-fatal outcomes related to cardiovascular diseases (CVDs). Additionally, recent evidence has demonstrated the association between hair mercury and dyslipidemia and non-communicable diseases [17,18,19]. However, only one study [42] since 2015 was found in our search that evaluated polymorphisms related to CVD (specifically the endothelial nitric oxide synthase (eNOS) gene). This preliminary evidence demonstrated that the 27-bp variable-number tandem repeat (VNTR) in intron 4 (4b/4a + 4a/4a) may be associated with decreased diastolic blood pressure and be a protective factor in exposed populations. Future research must be devoted to better understand the genetic modulation of the cardiovascular consequences of mercury exposure, especially considering the deleterious outcomes even with relatively low exposure.

This review presents some limitations. The search focused on a recent time range (since 2015) and consequently did not include previous evidence on genetic modulation of mercury exposure and intoxication. Additionally, genetic studies without mercury quantitation were not included. Unfortunately, the diversity of the polymorphisms and populations of the included studies did not allow is to perform meta-analyses. 

However, this review has some strengthens. The systematic search in three different databases and the following of guidelines guarantees the quality of this review. The analysis of recent studies was deliberate, since previous narrative reviews [24,30] had already covered previous knowledge. Here, we focused on understanding what was missing in recent research, aiming to provide insights for future directions. In this context, the quantitation of mercury was essential for two main reasons: (i) understanding the exposure context in which the possible genetic modulation (or its absence) was found and (ii) analyzing the modulation of mercury levels (toxicokinetic modulation) and possible modulation of the mercury effects (toxicodynamic modulation). This allows us to suggest an important insight: the possible biphasic behavior for the genetic modulation of mercury outcomes according to the exposure context, at least in some cases. Moreover, recommendations such as evaluating the possible ancestry effect in the analysis of genetic modulation, increasing the number of epidemiological studies with populations from the Southern Hemisphere or analyzing polymorphisms related to the cardiovascular consequences of the chronic exposure to mercury, among others, are proposed.

## 4. Conclusions

This scoping review systematically evaluated, for the first time, the genetic susceptibility to mercury exposure and intoxication using single nucleotide polymorphism analyses and mercury quantitation to obtain a clear view of the current scenario on this topic. Although there is still a long way to go towards personalized medicine in the prevention and treatment of mercury intoxication, the advances in genetics and sequencing have revolutionized the diagnosis and treatment of many diseases over recent years; it is therefore imperative to keep up with the scientific advances. By performing a systematic search in high-quality databases, this review allowed some important insights and recommendations for future studies, in addition to the careful analysis of the most recent results, thus definitely contributing to improve future research on the genetic modulation of mercury toxicity.

## Figures and Tables

**Table 2:** Highlights of the significant associations found between SNPs and mercury levels/outcomes.

Main Exposure	Matrix	Study	Main Significant Associations
Occupational	Blood Urine	[49]	Individuals showing CC-HSPA1A (+190G/C) and GG-HSPA1B (+1267A/G), alone or in combination, have a high predicted risk of developing chronic mercury poisoning.
	Urine	[27]	The SNPs ABCC2 (rs1885301, rs2273697) may differently modulate the individual performance of exposed individuals in neurological tests depending on ancestral background: in African populations, A allele carriers (rs1885301) showed significantly worse performance on the pencil tapping test; in African and Asian populations: A-allele carriers (rs2273697) showed a significantly better performance than GG carriers on the pencil tapping test.
	Blood Urine Hair	[50]	The G allele carriers for SLC22A8 (rs4149182) and the T allele carriers for ABCB1 (rs1202169) had an increased urinary clearance rate for mercury. The A allele carriers for SLC22A6 (rs4149170) and the C allele carriers for ABCB1 (rs1202169) showed abnormal levels of estimated glomerular filtration rate and beta-2-microglobulin.
	Blood Urine Hair	[29]	The T allele carriers for GCLM (rs41303970) were associated with higher urinary clearance rate of mercury. The C allele carriers for GCLC (rs1555903) were associated with lower levels of beta-2-microglobulin in the exposed group. An interaction between GSTA1 C allele (rs3957356) and GSS G allele (rs3761144) was associated with higher urinary levels of mercury in the exposed group.
Occupational and environmental	Hair Blood Urine	[47]	Multivariate analyses with Bonferroni corrections showed that heterozygotes and minor homozygotes of MT1M rs2270836, MT4 rs11643815 and GCLC rs138528239 accumulated more mercury in high consumers of fish. However, heterozygotes and minor homozygotes of ATP7B rs1061472 and rs732774 and BDNF rs6265 accumulated less mercury in high consumers of fish.
Environmental	Blood	[41]	GSTM1 deletion, ALAD1/2 (rs1800435) and A allele carriers for VDR (rs1544410) had higher Hg concentrations in the blood.
		[44]	G allele carriers for MT1A (rs8052394) in the third tertile of blood mercury showed significantly lower total and attention score.
		[37]	GSTT1 deletion was associated with reduced placental transfer of mercury.
		[59]	The SNP GSTP1 (rs1695, A allele) was associated with high concentrations of blood mercury.
	Hair	[51]	The SNPs ABCC1 (rs11075290, T allele; rs212093, G allele; and rs215088, G allele), ABCC2 (rs717620, T allele) and ABCB1 (rs10276499, T allele; rs1202169, C allele; and rs2032582, T allele) were associated with increased mercury concentration in maternal hair. The SNP ABCC1 (rs11075290, C allele) was associated with poorer performance in childhood neurodevelopment.
		[36]	In individuals showing ≥10 µg/g of total mercury in hair, E4 allele carriers for APOE (rs429358, rs7412) had higher levels than E2 allele carriers.
		[55]	The SNPs GCLC-129 (rs17883901, A allele), GPX1-198 (rs1050450, T allele) and MT1M (rs9936741, C allele) were associated with significantly lower hair mercury levels in mothers. The SNPs GSTP1 (rs1695, G allele) and MT1M (rs2270836, T allele) were associated with higher maternal hair mercury concentrations.
		[28]	With an increase in mercury levels, children carrying the GSTP1 rs1695 GA or GG alleles scored worse on problems such as anxiety, depression and somatic complaints than children with the AA genotype. The presence of the E4 allele for APOE (rs7412) was associated with signs such as anxiety, depression, somatic complaints, and social, thought and attention problems in exposed children.
	U cord	[56]	The SNP ABCC1 (rs11075290, T allele) was associated with decreased umbilical cord mercury concentrations.
	Hair U cord	[38]	E4 allele carriers for APOE (rs429358, rs7412) were associated with decreased cognitive performance in children.
		[61]	In girls, the BDNF SNPs rs7934165 GA, rs7103411 TT, rs11030104 AA and rs6265 CC were associated with lower estradiol levels with increasing cord blood mercury concentrations. In boys, the BDNF SNPs rs6265 CC and rs11030104 AA genotypes were associated with higher testosterone levels with increasing cord blood mercury concentrations.
	Blood Hair Milk U cord Urine	[54]	E4 allele carrier mothers had significantly higher mean levels of (methyl)mercury in peripheral venous blood, cord blood and hair.
	Blood Hair U cord	[52]	The SNP GCLC (rs761142, T allele) polymorphism and the combination of GCLC (rs761142, TT) and GCLM (rs41303970, CC) were associated with increased maternal hair mercury concentrations. Additionally, maternal GSTP1 (rs1695, G allele) was associated with a poorer neurodevelopmental performance in children.
